# Elevated expression of the RNA-binding protein IGF2BP1 enhances the mRNA stability of INHBA to promote the invasion and migration of esophageal squamous cancer cells

**DOI:** 10.1186/s40164-023-00429-8

**Published:** 2023-08-29

**Authors:** Juan-Juan Wang, Ding-Xiong Chen, Yu Zhang, Xin Xu, Yan Cai, Wen-Qiang Wei, Jia-Jie Hao, Ming-Rong Wang

**Affiliations:** 1 State Key Laboratory of Molecular Oncology, Center for Cancer Precision Medicine, National Clinical Research Center for Cancer/Cancer Hospital, National Cancer Center, Chinese Academy of Medical Sciences (CAMS), Peking Union Medical College (PUMC), 17 Panjiayuan Nanli, Chaoyang District, Beijing, 100021 China; 2 Stem cell Translational laboratory, Shanxi Technological Innovation Center for Clinical Diagnosis and Treatment of Immune and Rheumatic Diseases, Tongji Shanxi Hospital, Shanxi Bethune Hospital, Shanxi Academy of Medical Sciences, Third Hospital of Shanxi Medical University, Taiyuan, 030032 China; 3 Department of Cancer Epidemiology, National Clinical Research Center for Cancer/Cancer Hospital, National Cancer Center, Chinese Academy of Medical Sciences and Peking Union Medical College, Beijing, 100021 China

**Keywords:** Esophageal squamous cell carcinoma, Migration, Invasion, RNA binding protein, IGF2BP1, INHBA

## Abstract

**Background:**

The mechanisms underlying the occurrence and development of esophageal squamous cell carcinoma (ESCC) remains to be elucidated. The present study aims to investigate the roles and implications of IGF2BP1 overexpression in ESCC.

**Methods:**

IGF2BP1 protein expression in ESCC samples was assessed by immunohistochemistry (IHC), and the mRNA abundance of IGF2BP1 and INHBA was analyzed with TCGA datasets and by RNA in situ hybridization (RISH). The methylation level of the *IGF2BP1* promoter region was detected by methylation-specific PCR (MSP-PCR). Cell viability, migration, invasion and in vivo metastasis assays were performed to explore the roles of IGF2BP1 overexpression in ESCC. RNA immunoprecipitation sequencing (RIP-seq) and mass spectrometry were applied to identify the target RNAs and interacting proteins of IGF2BP1, respectively. RIP-PCR, RNA pulldown, immunofluorescence (IF), gene-specific m^6^A PCR and RNA stability assays were used to uncover the molecular mechanisms underlying the malignant phenotypes of ESCC cells caused by IGF2BP1 dysregulation. BTYNB, a small molecular inhibitor of IGF2BP1, was evaluated for its inhibitory effect on the malignant phenotypes of ESCC cells.

**Results:**

IGF2BP1 overexpression was detected in ESCC tissues and associated with the depth of tumor invasion. In addition, IGF2BP1 mRNA expression in ESCC cells was negatively correlated with the level of its promoter methylation. Knockdown of IGF2BP1 inhibited ESCC cell invasion and migration as well as tumor metastasis. Mechanistically, we observed that IGF2BP1 bound and stabilized INHBA mRNA and then resulted in higher protein expression of INHBA, leading to the activation of Smad2/3 signaling, thus promoting malignant phenotypes. The mRNA level of INHBA was upregulated in ESCC tissues as well. Furthermore, IGF2BP1 interacted with G3BP stress granule assembly factor 1 (G3BP1). Knockdown of G3BP1 also down-regulated the INHBA-Smad2/3 signaling. BTYNB abolished this activated signaling and significantly attenuated the malignant phenotypes of ESCC cells.

**Conclusions:**

Elevated expression of IGF2BP1 is a frequent event in ESCC tissues and might be a candidate biomarker for the disease. IGF2BP1 overexpression promotes the invasion and migration of ESCC cells by activating the INHBA-Smad2/3 pathway, providing a potential therapeutic target for ESCC patients with high expression of IGF2BP1.

**Supplementary Information:**

The online version contains supplementary material available at 10.1186/s40164-023-00429-8.

## Background

Esophageal cancer is one of the major malignancies threatening human health. Esophageal squamous cell carcinoma (ESCC) accounts for more than 90% of esophageal cancer cases in China. Most patients with ESCC are diagnosed at an advanced stage, and the overall 5-year survival rate is only approximately 30% [1] due to invasive growth and distal metastasis. However, the molecular mechanisms underlying the invasion and metastasis of ESCC are still not fully understood, and there are no effective targeted drugs for clinical treatment to date. Therefore, there is an urgent need to identify the key molecules affecting the invasion and metastasis of ESCC.

Insulin-like growth factor 2 mRNA binding protein 1 (IGF2BP1) is a highly conserved RNA binding protein that mainly binds mRNA and thereby affects RNA transcription, processing, translation and metabolism. IGF2BP1 overexpression is often correlated with poor prognosis in a variety of cancer types, including melanoma [2], breast [3], ovarian [4–6], colon [7, 8], liver [9, 10], and lung [11, 12] cancers. It has been reported that IGF2BP1 promotes cell proliferation or invasion by stabilizing several mRNA targets such as CD44 and c-myc [13], which have been confirmed as oncogenes [14, 15]. Moreover, IGF2BP1 has been proven to be a N6-methyladenosine (m^6^A) reader that recognizes and binds m^6^A-modified mRNA and thus enhances its stability [16–19]. Additionally, IGF2BP1 has been designated an oncofetal protein due to its space-time specific expression pattern: it is predominantly expressed in embryonic development and suppressed in most adult tissues but re-expressed in multiple tumor types [20].

We found remarkable upregulation of IGF2BP1 in ESCC tissues by immunohistochemistry (IHC). However, there is no information available on the role of IGF2BP1 in ESCC. In this study, we focused on the roles of IGF2BP1 overexpression in malignant phenotypes and the underlying mechanisms in ESCC cells, aiming to explore the possibility of IGF2BP1 as a biomarker and therapeutic target for the disease.

## Materials and methods

### Tissue specimens and cell lines

ESCC and operative margin tissues were procured from surgical resection specimens. All of the patients received no treatment prior to surgery and signed separate informed consent forms for sample collection and molecular analysis. The study was approved by the Ethics Committee of the Cancer Institute (Hospital), Chinese Academy of Medical Sciences (CAMS) & Peking Union Medical College (PUMC) (No. 16–171/1250).

ESCC cell lines KYSE30, KYSE70, KYSE150, KYSE180, KYSE450 and KYSE510 were generously provided by Prof. Shimada (Kyoto University, Japan); TE1, TE4 and TE10 were purchased from ATCC, and Eca109 was purchased from Cell Resource Center, Institute of Basic Medicine, Chinese Academy of Medical Sciences. All of the cell lines were authenticated by short tandem repeat (STR) profiling and cultured in RPMI-1640 media with 10% fetal bovine serum (FBS500-S, AusGeneX) in a humidified incubator at 37 °C and 5% CO_2_.

### Plasmid constructs, transfection and lentiviral transduction

The materials and methods are provided in Additional file 1: Table S1-3.

### Immunohistochemistry (IHC)

IHC assays were conducted as reported previously [21]. Slides were incubated with primary antibody at 4 °C overnight. The tissues were incubated with a Mouse/Rabbit Enhanced Polymer Detection System (PV-9000, ZSGB-BIO) and then chromogenic substrate DAB (ZLI-9017, ZSGB-BIO). The tissue microarrays were scanned with a Nano Zoomer digital pathology biopsy scanner (HAMAMATSU, Japan). Immunoreactive scores were calculated by multiplying the scores of staining signal intensity and the percentage of positive cells. The intensity was scored as follows: 0 (negative), 1 (weak), 2 (moderate), and 3 (strong); the proportion of positive cells was scored as follows: 0 (negative), 1 (1-20%), 2 (21-50%), and 3 (51-100%). Antibodies used for IHC were listed in Additional file 1: Table S4.

### RNA in situ hybridization (RISH)

INHBA mRNA in situ hybridization was performed on 6 μm thick tissue microarrays (TMAs) with RNAscope 2.5 HD Reagent Kit-BROWN (322,300, ACD) following the manufacturer’s instructions.

### Cell viability and colony formation assay

1 × 10^3^ cells were seeded into each well of a 96-well plate (with 3 replicates in each group), and the cell viability was quantified every 24 h using Cell Counting Kit-8 (CK-04, Dojindo, Japan) according to the manufacturer’s instructions. Absorbance at 450 nm was measured by an Elx 808 Microplate Reader (BioTek, USA). For the colony formation assay, 1 × 10^3^ cells were seeded into each well of a 6-well plate and treated with the indicated dose of BTYNB (with 3 replicates in each group) for 7 ~ 14 days. The colonies of cells were fixed with 100% methanol, stained using 0.1% crystal violet, and then counted.

### Wound-healing assay

Cells were seeded in six-well plates and grown until they reached full confluence. Cells were scratched a wound vertically and washed with PBS. The scratches were observed and photographed at indicated time points. The wound areas were measured using ImageJ (Ver. 1.51j8, NIH, USA).

### Cellular invasion and migration assays

Invasion and migration assays were performed in Transwell plates as described previously [22]. After incubation for 36 h (KYSE30) or 24 h (TE1), the membranes with stained cells were placed on slide and mounted with coverslip, followed by scanning and imaging with a Nano Zoomer digital pathology biopsy scanner (HAMAMATSU, Japan). The areas covered by stained cells in three random fields were measured by ImageJ. More details are provided in Additional file 1: Supplementary_Materials and Methods.

### Cell apoptosis analysis

Cells treated with 10 µM BTYNB for 48 h were digested, collected and stained with fluorescently labeled Annexin V and PI using an Annexin V FITC Apoptosis Detection Kit (AD10, Dojindo). Flow cytometry was adopted to detect the percentage of apoptotic cells.

### Xenograft assay

Four-week-old female BALB/c nude mice (HFK Bioscience Co., LTD, Beijing, China) were purchased and randomly divided into two groups by body weight (10 per group). The mice were injected with 1 × 10^6^ KYSE30 cells stably expressing shIGF2BP1 or shNon-silencing (shNS) via the tail vein. Eight weeks later, the mice were sacrificed, and the whole lung tissues were separated and fixed in Bouin’s Fixative Solution (PH0976, Phygene). Then, the number of lung metastases was counted, and the lung tissues were embedded in paraffin, cut into 3 μm sections, and stained with hematoxylin and eosin (H&E). All animal experiments were approved by the Animal Center of the Institute of National Cancer Center/Cancer Hospital, CAMS & PUMC (NCC2019A016).

### Western blotting

Total protein was isolated using RIPA buffer (C1053, Applygen) supplemented with protease inhibitors (B14001, Bimake) and phosphatase inhibitors (B15001, Bimake) and quantified with a Pierce BCA Protein Assay Kit (23,225, Thermo). Antibodies for immunoblotting were listed in Additional file 1: Table S4.

### Reverse transcription PCR (RT-PCR) and quantitative real-time RT-PCR (qRT-PCR)

Total RNA was isolated using an RNApure Tissue & Cell Kit (CW0506, Cwbiotech) following the manufacturer’s instructions, and cDNA was synthesized using a HiFiScript cDNA Synthesis Kit (CW2569M, Cwbiotech). Then, RT-PCR was conducted with TaKaRa Ex Taq (RR001A, TaKaRa) on a SimpliAmp Thermal Cycler (ABI, USA). qRTPCR was performed using a TB Green™ Premix Ex Taq Kit (RR420A, TaKaRa) on an ABI QuantStudio DX real-time PCR system (ABI, USA). The relative expression levels of mRNA were assessed through the comparative threshold cycle method (2^−ΔΔCt^) with GAPDH as an internal control. All primers used in this study are listed in Additional file 1: Table S5.

### RNA coimmunoprecipitation combined with high-throughput sequencing (RIP-seq)

RIP was performed using an EZ-Magna RIP Kit (17–701, Millipore). RNA was finally purified with TRIzol reagent (Invitrogen) and analyzed by RT-PCR or RNA-seq (Wuhan Seqhealth Tech Co. Ltd.). The sequences of primers for RT-PCR are described in Additional file 1: Table S5.

### Biotin RNA pull-down assay

RNA pull-down assays were performed as previously described [23]. The RNA-protein complex was immunoprecipitated with streptavidin magnetic beads (HY-K0208, MedChemExpress). The complex was divided into two equal portions for RT-PCR and WB analysis. The sequences of biotin-labeled DNA probes are provided in Additional file 1: Table S6.

### RNA stability assay

Cells were treated with actinomycin D (ActD, 5 µg/mL) for 0, 2, or 4 h. Total RNA was extracted, and the relative level of mRNA at each time point was analyzed by quantitative real-time PCR with GAPDH as an internal control. The mRNA half-life was estimated according to a previous description[24]. Primers for qPCR are listed in Additional file 1: Table S5.

### Gene-specific m^6^A qPCR

The methylated mRNAs were immuno-precipitated as previously reported [25], eluted with elution buffer (10 mL of 0.1 M DTT, 0.44 g of NaCl, 2.5 mL of pH 7.5 1 M Tris-HCl, 0.1 mL of 0.5 M EDTA, 0.5 mL of 10% SDS, 10 µL of RNase inhibitor, ddH_2_O up to 50 mL) and recovered with the RNeasy Micro Kit (74,004, Qiagen), further analyzed by RT–PCR along with input control. m^6^A antibody was described in Additional file 1: Table S4.

### Methylation-specific PCR (MSP-PCR)

Genomic DNA of ESCC cells was extracted using the QIAamp DNA Mini Kit (QIAGEN) and transformed with the Epitect Fast DNA Bisulfite Kit (QIAGEN). The sequences of primer pairs against the first intron are provided in Additional file 1: Table S7.

### Coimmunoprecipitation-based mass spectrometry (Co-IP-MS)

Protein A/G magnetic beads (HY-K0202, MedChemExpress) were used to perform coimmunoprecipitation. Protein samples were then subjected to WB assay or SDS–PAGE followed by Coomassie staining. Gel pieces were cut off and sent to Shanghai Applied Protein Technology Co. Ltd. for mass spectrometry analysis. Antibodies used for Co-IP were listed in Additional file 1: Table S4. More details are provided in Additional file 1: Supplementary _Materials and Methods.

### Immunofluorescence (IF) staining

Immunofluorescence was performed conventionally and the antibodies were described in Additional file 1: Table S4. Immunofluorescence was detected by confocal microscopy (PE double spinning disk confocal, USA).

### Database analysis

The Cancer Genome Atlas (TCGA) datasets [26] (https://tcga.xenahubs.net) and the Wanderer interactive viewer [27] (http://maplab.imppc.org/wanderer/) were employed to explore the mRNA expression of IGF2BP1 and INHBA in squamous cell carcinoma (SCCs), invasive breast cancer and corresponding normal tissues; the expression of IGF2BP1 mRNA in all major tissues and organs in the human body was analyzed with the HPA database [28] (https://www.proteinatlas.org/).

### Statistical analysis

IBM SPSS Statistics 23.0 software was applied for data analysis, and *P* < 0.05 was considered statistically significant. Fisher’s exact test was used to assess the IHC score difference between ESCC tissues and adjacent noncancerous specimens. The correlation between the protein expression level and clinicopathological parameters was analyzed by Pearson’s chi-square test. Comparisons between two groups were performed by independent samples T tests, and one-way ANOVA was used for multiple comparisons. Rstudio software (1.1463) was used for Gene Ontology (GO) and pathway enrichment analysis.

## Results

### IGF2BP1 is highly expressed in ESCC tissues and is associated with the depth of tumor invasion

**Fig. 1:**
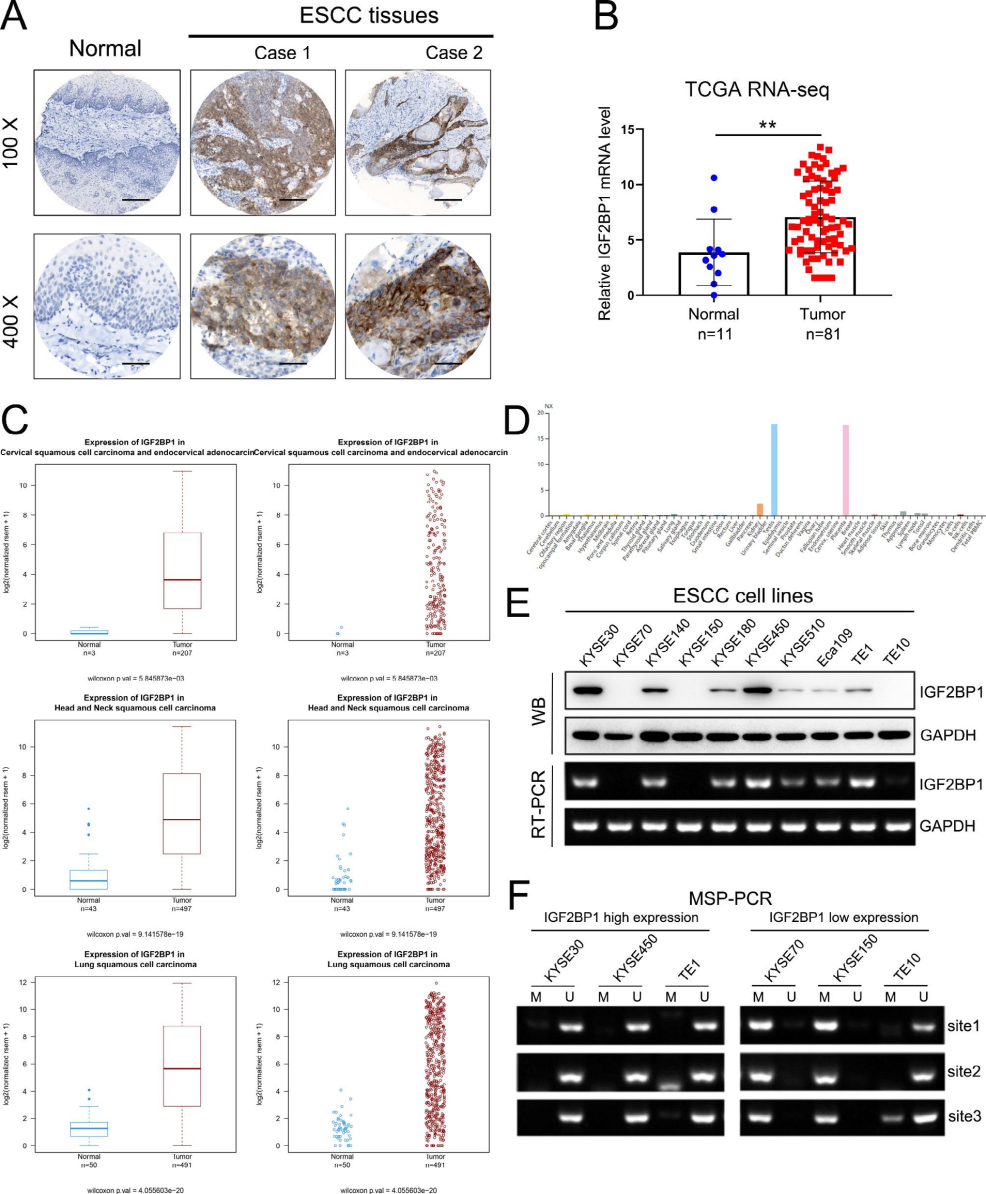
IGF2BP1 is overexpressed in SCC tissues. (**A**) Representative IHC staining of IGF2BP1 in ESCC and adjacent nonmalignant tissues. Scale bar = 200 μm (100×); scale bar = 50 μm (400×). (**B**) IGF2BP1 mRNA levels in ESCC patients and normal tissues in TCGA database. **P < 0.01. (**C**) IGF2BP1 mRNA levels in HNSCC, LUSC, CESC patients and normal tissues in TCGA database. (**D**) The expression of IGF2BP1 mRNA in all major tissues and organs in the human body was analyzed in the HPA database. (**E**) IGF2BP1 protein and mRNA levels of ESCC cell lines were analyzed using RT-PCR and Western blotting, respectively. (**F**) The methylation level of the first intron of the IGF2BP1 gene in the ESCC cell genome was assessed by MSP-PCR. Three pairs of methylated (M) and unmethylated (U) primers targeting three CG sites in the first intron were designed to amplify DNA converted by bisulfite

We examined the protein expression level of IGF2BP1 in 311 ESCC tissues and 9 adjacent normal tissues by IHC. The results showed that IGF2BP1 was highly expressed in ESCC tissues (155/311, 49.8%) but was not expressed or only weakly expressed in normal esophageal epithelia (Fig. 1A; Table 1). Positive staining was predominant in the cytoplasm of ESCC cells. A higher IGF2BP1 expression level was positively correlated with the depth of tumor invasion (T_1 − 2_ versus T_3 − 4_), but no significant differences were found in other clinicopathological features, such as sex, age, histologic grade, lymph node metastasis and clinical stage (Table 1). Furthermore, an analysis of RNA-seq data obtained from the TCGA database revealed that the mRNA expression level of IGF2BP1 was elevated in ESCC specimens compared with normal tissues (Fig. 1B), which was consistent with the IHC results.

We further found higher mRNA expression of IGF2BP1 in other SCCs (Head and neck squamous cell carcinoma, HNSCC; Lung squamous cell carcinoma, LUSC; Cervical squamous cell carcinoma, CESC) tissues than in the corresponding normal tissues based on TCGA datasets (Fig. 1C). Interestingly, according to the HPA database, IGF2BP1 was almost absent in normal tissues except in the embryo and reproductive system (Fig. 1D). Moreover, we observed a high degree of consistency between the mRNA and protein levels of IGF2BP1 in 10 ESCC cell lines (Fig. 1E). To uncover the mechanism of IGF2BP1 mRNA upregulation in ESCC cells, we examined the methylation levels in the promoter region of this gene. The results showed that the first intron of IGF2BP1 gene was hypomethylated in cell lines with high IGF2BP1 expression but hypermethylated in cell lines with low IGF2BP1 expression, except for TE10 (Fig. 1F).

**Table 1:** The association between IGF2BP1 expression and the clinicopathological variables

Clinicopathological parameter	Case number	IGF2BP1 expression		
		Positive (%)	χ^2^	*P-*value
Age				
< 60	98	44 (44.90)	0.361	0.548
≥ 60	213	111 (52.10)		
Gender				
male	225	114 (50.70)	0.223	0.637
female	86	41 (47.70)		
Grade				
G1	44	16 (36.36)	3.724	0.155
G2	200	104 (52.00)		
G3	67	35 (52.24)		
pT				
T1-2	84	33 (39.29)	5.127	**0.024**
T3-4	227	122 (53.74)		
pN				
N0	161	81 (50.31)	0.03	0.863
N1-4	150	74 (49.33)		
Stage				
I	30	14 (46.67)	0.493	0.92
II	143	71 (49.65)		
III	120	62 (51.67)		
IV	18	8 (44.44)		

Statistical significance (*P* < 0.05) is shown in bold

### IGF2BP1 promotes ESCC cells invasion, migration in vitro and lung metastasis in nude mice

**Fig. 2:**
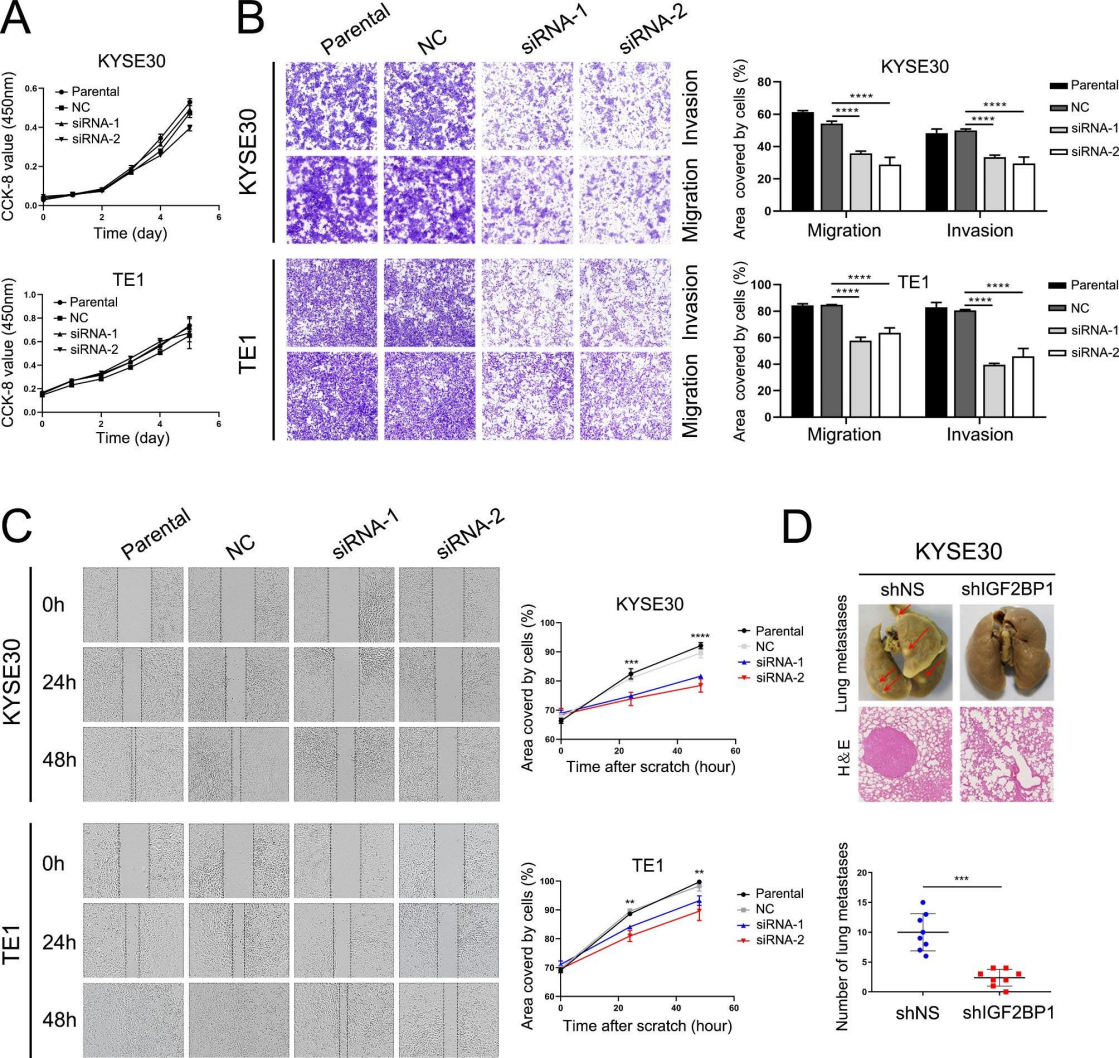
IGF2BP1 promotes the invasion and migration of ESCC cells in vitro as well as lung metastasis in vivo. (**A**) Cell viability was quantified using a CCK-8 assay. (**B**) Cell invasion and migration abilities were examined using Transwell assays. Representative results (left) and statistical plots (right) are shown. (**C**) Cell motility was assessed by the wound-healing assay. Representative results (left) and statistical plots (right) are shown. (**D**) Cell metastatic potential was evaluated using an in vivo pulmonary metastasis assay. Representative images of fixed lung tissues (top) and the results of H&E staining (bottom) are shown. The arrows indicate the lung metastatic nodules. The number of metastatic nodules was plotted (right). **P < 0.01; ***P < 0.001; ****P < 0.0001. NC: Negative Control

To identify the role of IGF2BP1 in ESCC, KYSE30 and TE1 with high IGF2BP1 expression were applied for functional study as cell models (Fig. 1E). After transient knockdown of IGF2BP1 mediated by small interfering RNA (siRNA), there was no significant change in cell growth within six days compared with the negative control group (Fig. 2A). However, the migration and invasion ability of both cell lines were substantially repressed after IGF2BP1 silencing in the Transwell assays (Fig. 2B) and wound healing assays (Fig. 2C).

Next, KYSE30 cells stably expressing shRNA of IGF2BP1 (shIGF2BP1) or nonsilencing shRNA (shNS) conducted by lentivirus infection were injected into nude mice via the tail vein. The formation of lung metastatic tumors was observed in both groups six weeks later, but the number of lung metastases in the shIGF2BP1 group was significantly reduced, and the metastatic nodules were noticeably smaller (Fig. 2D).

### IGF2BP1 increases INHBA mRNA stability, promoting ESCC cells invasion and migration

**Fig. 3:**
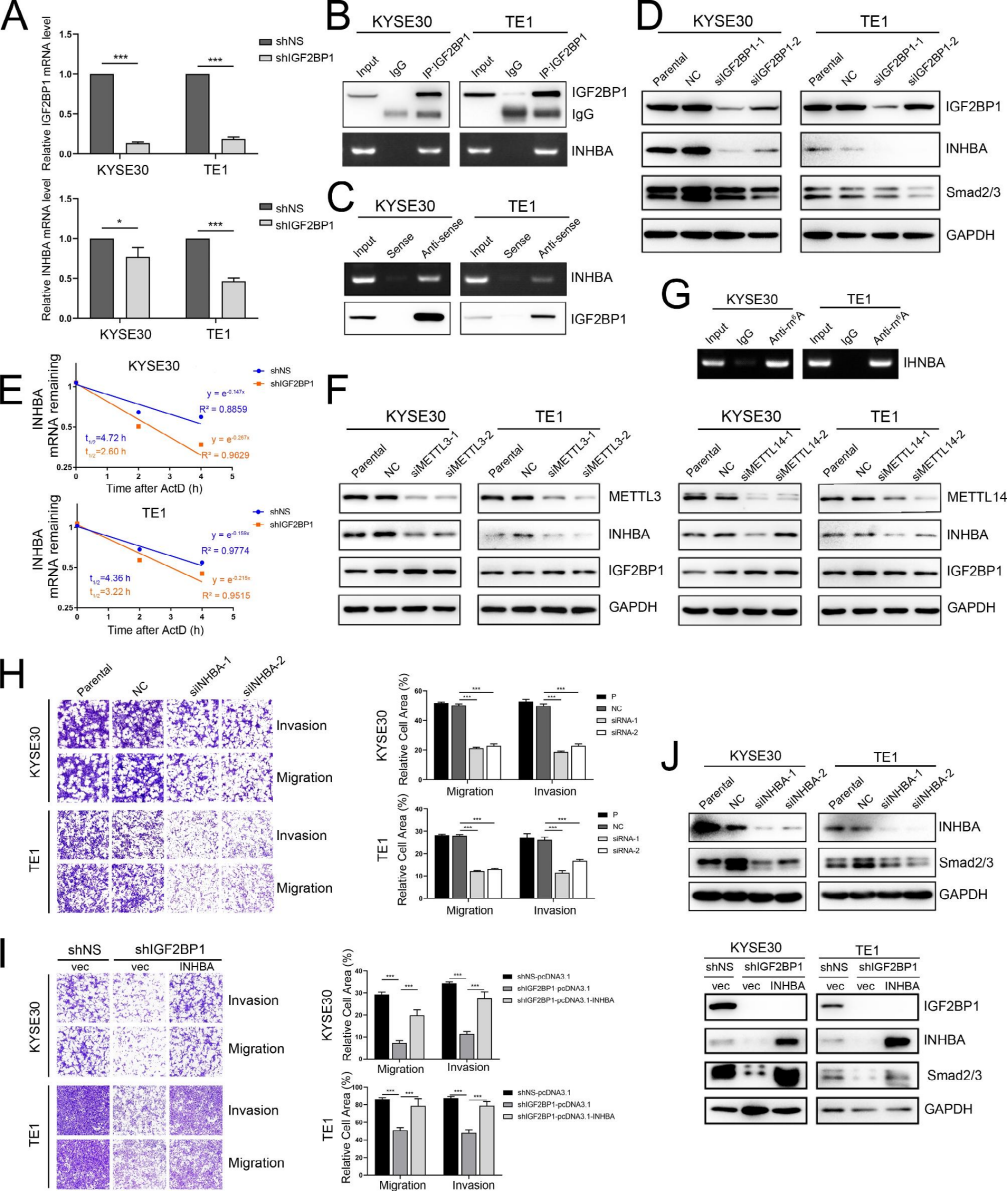
IGF2BP1 enhances ESCC cell invasion and migration by activating INHBA-Smad2/3 signaling. (**A**) The mRNA levels of IGF2BP1 and INHBA in KYSE30 and TE1 cells after IGF2BP1 knockdown were determined by qRT-PCR. (**B-C**) The interaction between IGF2BP1 protein and INHBA mRNA in ESCC cells was validated with RIP-PCR (B) and RNA pull-down assay (C). (**D**) Western blotting analysis of the indicated proteins in ESCC cells transfected with IGF2BP1-specific siRNA or NC siRNA. (**E**) The decay rate of INHBA mRNA after IGF2BP1 depletion was evaluated by RNA stability assay. (**F**) Cell lysates were immunoblotted for the indicated proteins after METTL3/14 transient knockdown. (**G**) m^6^A modification in INHBA mRNA was tested by gene-specific m^6^A PCR. (**H-I**) Cell invasion and migration abilities were examined with Transwell assays. (**J**) Western blotting analysis of INHBA and Smad2/3 in ESCC cells transfected with INHBA siRNA (above) and in ESCC cells stably expressing shIGF2BP1 transfected with pcDNA3.1-INHBA or empty vector (below). *P < 0.05; ***P < 0.001

Given that IGF2BP1 is an RNA binding protein, we performed RIP-seq in KYSE30 cells to identify its potential RNA targets. RIP-seq profiling revealed that most of the IGF2BP1-binding sites were highly enriched in coding regions (CDSs) and 5’ untranslated region (5’-UTR) of its mRNA targets.(Additional file 2: Fig. S1). Through GO analysis of the identified results (log2 FC > 1 and FDR < 0.01) by RIP-seq, we selected 30 genes related to cell invasion and migration as candidates (Additional file 3: Table S8 and S9). Then, qRT-PCR was employed to detect the mRNA expression of the candidate genes in IGF2BP1 stable knockdown KYSE30 and TE1 cell lines. Notably, the reduction in INHBA mRNA abundance after IGF2BP1 knockdown was most significant among the 30 candidate genes in KYSE30 and TE1 cells (Fig. 3A, Additional file 3: Table S10, Additional file 4: Fig. S2, Additional file 5: Fig. S3). RIP-PCR and RNA pulldown using biotin-labeled DNA probes against INHBA mRNA further confirmed the interaction between IGF2BP1 protein and INHBA mRNA (Fig. 3B, C). Furthermore, silencing IGF2BP1 downregulated the protein expression of INHBA and Smad2/3 in KYSE30 and TE1 cells (Fig. 3D).

Previous studies have shown that IGF2BP1 can recognize N6-methyladenosine (m^6^A) and enhance mRNA stability and translation in an m^6^A-dependent manner [16, 17]. Therefore, it is highly possible that IGF2BP1 binds and stabilizes the mRNA of INHBA. RNA stability assays showed that the half-life of INHBA mRNA was significantly shortened after knockdown of IGF2BP1 in KYSE30 and TE1 cells (Fig. 3E). As the m^6^A writers METTL3 or METTL14 was repressed by siRNAs, the INHBA protein levels were decreased in both cell lines without affecting IGF2BP1 (Fig. 3F). Moreover, gene-specific m^6^A qPCR further confirmed that INHBA was regulated by m^6^A modification (Fig. 3G).

INHBA is a member of the transforming growth factor β (TGF-β) superfamily, which is closely associated with tumor invasion and metastasis. We then assessed the effects of INHBA on invasive and migratory phenotypes in ESCC cells. Cell invasion and migration were inhibited after knockdown of INHBA mediated by siRNA (Fig. 3H), while transient overexpression of INHBA in cells with stable knockdown of IGF2BP1 partially overcame this suppression (Fig. 3I). Western blotting results showed that Smad2/3 expression was repressed by IGF2BP1 depletion in KYSE30 and TE1 cells, while the IGF2BP1 knockdown-induced Smad2/3 decrease was reversed by INHBA overexpression (Fig. 3J).

### IGF2BP1 interacts with G3BP1

**Fig. 4:**
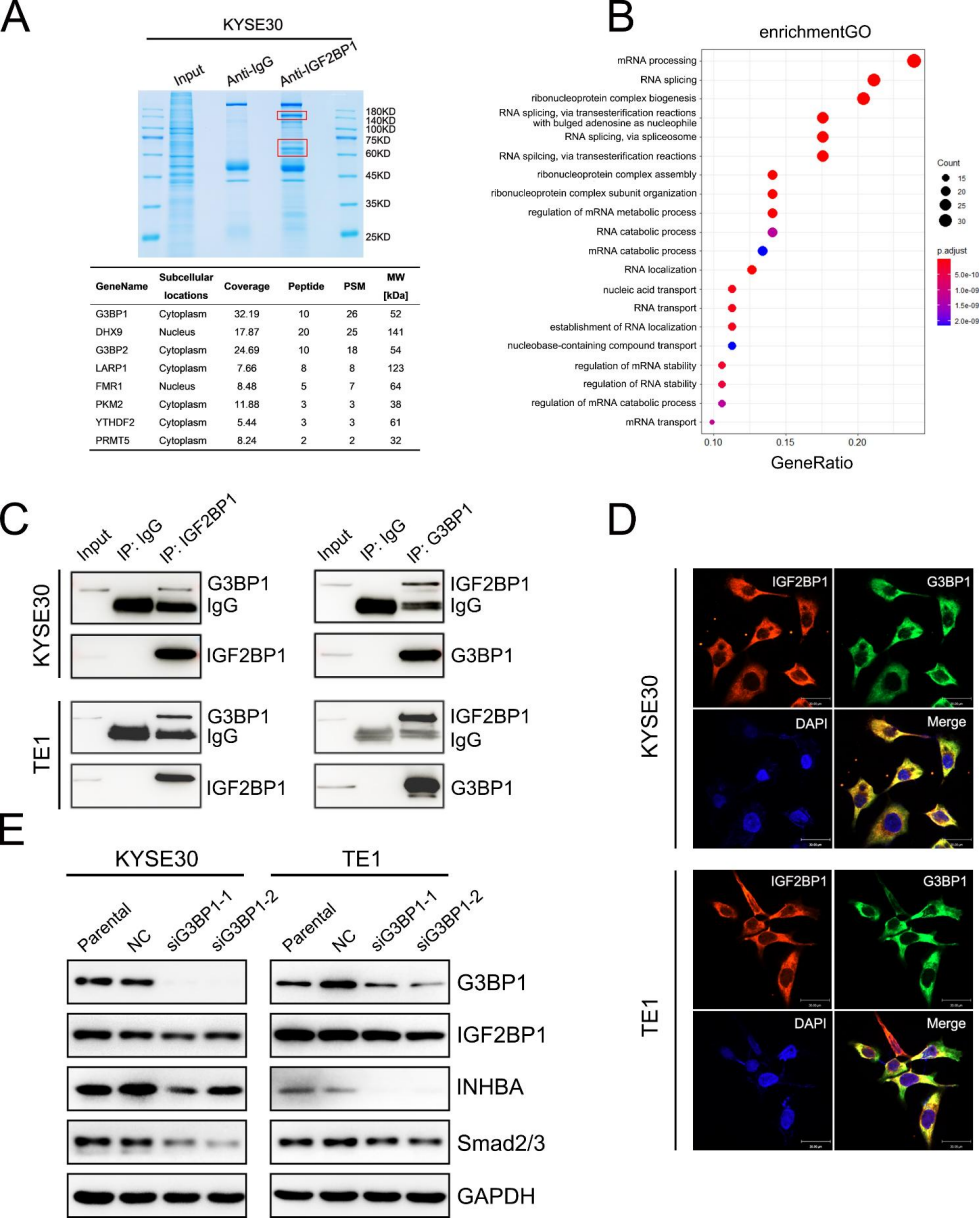
G3BP1 interacts with IGF2BP1. (**A**) Identification of interacting proteins by Co-IP-MS. The red boxes indicate the differential proteins that were cut off and identified using MS. The whole lane of the IgG group served as a negative control. The table below shows the top eight candidate interacting proteins. (**B**) Bubble plot of GO enrichment based on mass spectrometry results. (**C**) The interaction between IGF2BP1 and G3BP1 in ESCC cells was detected with an endogenous immunoprecipitation assay. (**D**) Cellular localization of endogenously expressed IGF2BP1 (red) or G3BP1 (green) was detected by immunofluorescence staining using laser confocal microscopy. DAPI was used to stain nuclei (blue). Scale bar = 30 μm. (**E**) Western blotting analysis of the indicated proteins in ESCC cells transfected with NC-siRNA or G3BP1 siRNA. GAPDH was used as a loading control

It has been reported that IGF2BPs interact with other RNA-binding proteins (RBPs) to regulate mRNA targets [29, 30]. To further elucidate the functional mechanism of IGF2BP1 in ESCC cells, we adopted Co-IP-MS to investigate interactive partners of IGF2BP1 (Fig. 4A). A total of 227 potential proteins were identified (Additional file 6: Table S11), and 46% of them participate in RNA regulation (Additional file 6: Table S12). GO enrichment analysis revealed that most of them were involved in mRNA processing, ribonucleoprotein complex biogenesis, RNA splicing, regulation of mRNA stability (Fig. 4B).

We selected RNA-binding proteins as candidates for validation according to the following criteria: (1) the candidates are related to invasion and migration by GO analysis; (2) the sub-cellular localization of candidates are in the cytoplasm as IGF2BP1. Based on the above criteria, we got eight candidate proteins among which G3BP1 had the top Coverage and PSM values in the IP group (Fig. 4A, Additional file 6: Table S13). Thus, G3BP1 was selected for further study. The interaction between G3BP1 and IGF2BP1 was confirmed by endogenous Co-IP (Fig. 4C) and IF staining using confocal microscopy (Fig. 4D). Moreover, silencing G3BP1 led to a decrease of INHBA and Smad2/3, as well as a slight decrease of IGF2BP1 (Fig. 4E).

### INHBA-Smad2/3 is significantly upregulated in ESCC tissues

**Fig. 5:**
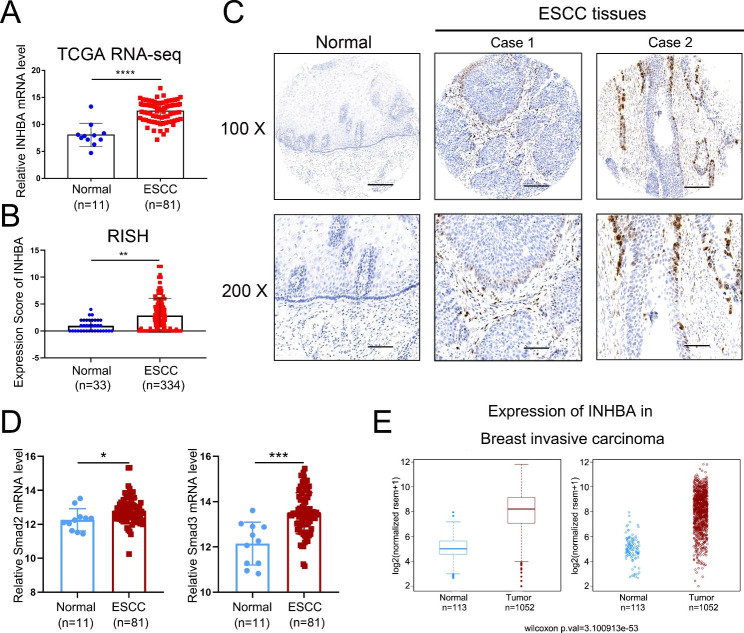
INHBA is upregulated in ESCC and invasive breast cancer. (**A**) INHBA mRNA levels in ESCC and normal tissues analyzed using TCGA datasets. (**B**) RISH scores of INHBA in ESCC and normal tissues. (**C**) Representative RISH staining of INHBA mRNA in ESCC and adjacent normal tissues. Scale bar = 200 μm (100×); scale bar = 100 μm (200×). (**D**) Analysis of Smad2 and Smad3 mRNA levels in ESCC and normal tissues in TCGA datasets. (**E**) INHBA mRNA levels in invasive breast cancer and normal tissues from TCGA database. *P < 0.05; **P < 0.01; ***P < 0.001; ****P < 0.0001

We then analyzed TCGA transcriptome sequencing datasets and observed that the mRNA of INHBA was also increased in ESCC tissues (Fig. 5A). RISH analysis of tissue microarrays further confirmed the RNA-seq results (Fig. 5B). INHBA mRNA was mainly distributed in the peripheral tumor cells of cancer nests and the stroma of ESCC tissues but was negative in the normal esophageal epithelia and stroma (Fig. 5C). Moreover, the mRNA abondance of Smad2 and Smad3, especially Smad3, was significantly increased in ESCC tissues compared with the normal controls based on TCGA datasets (Fig. 5D). Besides, in invasive breast cancer tissues, INHBA mRNA was significantly elevated (Fig. 5E).

### The small molecule inhibitor BTYNB significantly inhibits the invasion, migration and proliferation of ESCC cells in vitro

**Fig. 6:**
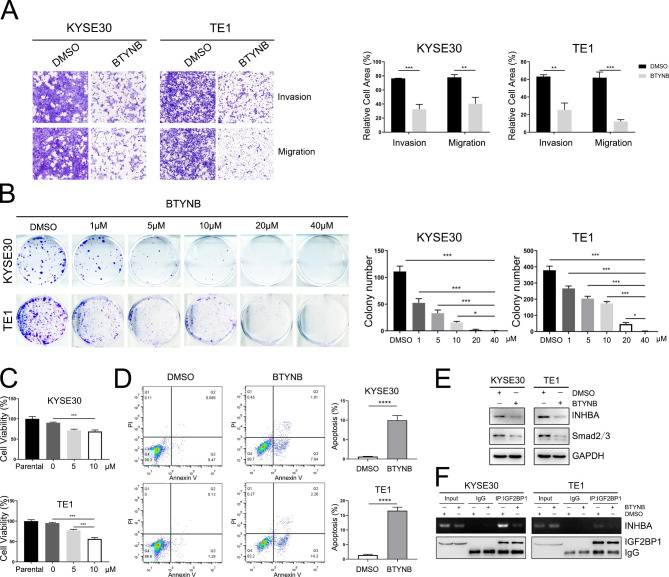
BTYNB inhibits the invasion, migration and proliferation of ESCC cells in vitro. (**A**) The effects of BTYNB on cell invasion and migration were examined by Transwell assay. 20 µM BTYNB was added to the lower compartment for 24-36 h. (**B**) Colony formation of KYSE30 and TE1 cells exposed to different concentrations of BTYNB. (**C**) Cell viability of ESCC cells exposed to BTYNB was determined by CCK-8 assay. KYSE30 and TE1 cells were treated with 5 µM and 10 µM BTYNB for 72 h. (**D**) Cell apoptosis was determined by flow cytometry. ESCC cells were treated with 10 µM BTYNB for 48 h. (**E**) Western blotting analysis of the indicated proteins in cells treated with BTYNB. (**F**) The IGF2BP1-INHBA interaction upon BTYNB treatment (20 µM BTYNB for 24 h) was assessed by RIP-PCR. *P < 0.05; **P < 0.01; ***P < 0.001; ****P < 0.0001

Currently, there are no inhibitors in clinical trials that directly target IGF2BP1. Previous studies reported that a small molecule drug, BTYNB, could inhibit the binding of IGF2BP1 to c-Myc mRNA [31, 32]. BTYNB also impairs cell proliferation in vitro by blocking β-TRCP1, E2F and other transcripts [32, 33]. Therefore, it is reasonable to speculate that BTYNB might interfere with the IGF2BP1-driven malignant phenotypes in ESCC cells. We tested BTYNB with a Transwell assay by adding the drug into the lower chamber and found that the invasion and migration of KYSE30 and TE1 cells were significantly inhibited at 36 h and 24 h, respectively (Fig. 6A). Meanwhile, colony formation and cell viability were impaired in a dose-dependent manner (Fig. 6B-C). In addition, 48 h of exposure to BTYNB increased the number of apoptotic cells (Fig. 6D). Western blotting analysis showed that INHBA and Smad2/3 expression was decreased after BTYNB treatment (Fig. 6E). RIP-PCR further implied that the IGF2BP1-INHBA interaction was disrupted by BTYNB in ESCC cells (Fig. 6F).

## Discussion

The insulin-like growth factor-2 mRNA-binding protein family (IGF2BPs), composed of IGF2BP1, IGF2BP2 and IGF2BP3, has a crucial role in early embryonic development. IGF2BP1 and IGF2BP3 are oncofetal proteins because they are mostly silent in adult organs, except in the reproductive system[13, 20, 34]. In contrast, IGF2BP2 is the only expressive IGF2BP in most adult tissues. IGF2BP1 and IGF2BP3 are re-expressed in many types of tumors, and IGF2BP2 was also found to be excessively expressed in malignancies due to genomic amplification according to pan-cancer analysis with TCGA data. Growing evidence supports the pro-oncogenic roles of these RNA-binding proteins in cancer progression by influencing their RNA target fate [35].

However, few studies on IGF2BP1 have been reported in ESCC. Herein, we discovered remarkably high expression of IGF2BP1 at both the mRNA and protein levels, indicating transcriptional dysregulation in ESCC. More importantly, our functional and mechanistic investigations revealed that IGF2BP1 facilitated the migration, invasion and metastasis of ESCC cells by activating the INHBA-Smad2/3 cascade. INHBA, a member of the TGF-β superfamily, has been reported to be overexpressed in multiple types of cancers, including ESCC, and associated with poor prognosis [36–41]. Consistently, our analyses of TCGA data and RISH on TMAs demonstrated the upregulation of INHBA at the transcriptional level in ESCC tissues. In addition, we found that INHBA mRNA was frequently overexpressed in ESCC and invasive breast cancer. More importantly, the downstream molecule of INHBA-Smad2 and Smad3 were also upregulated in ESCC tissues. Combined with the spatial distribution of INHBA mRNA in ESCC tissues, we speculate that INHBA may play an important role in cell invasion and migration. Although the roles of INHBA in cancer are controversial, the majority favor its oncogenic effects. Seder et al. reported that INHBA promoted cell proliferation and was regulated by promoter demethylation in ESCC cells [42]. Another study suggested that INHBA affects cell migration and positively correlated with genes involved in extracellular structure organization [43]. In the present study, we identified INHBA as a direct target of IGF2BP1 with a functional role in tumor invasion induced by IGF2BP1. Mechanistically, IGF2BP1 bound and stabilized INHBA mRNA, consequently leading to an increase in INHBA protein. Moreover, as an m^6^A reader proven by recent research, IGF2BP1 preferentially recognizes m^6^A-modified mRNAs and promotes their stability in an m^6^A-dependent manner [17]. We indeed observed that INHBA mRNA was m^6^A modified and that the turnover of INHBA was m^6^A-dependent. Therefore, it is likely that mRNA methylation is required in the regulation of INHBA by IGF2BP1.

RNA-binding proteins participate in forming ribonucleoprotein (RNP) granules that regulate mRNA translation, localization, and turnover [44]. Our Co-IP-MS results confirmed that IGF2BP1 functions by interacting with other RBPs. G3BP1 was validated as a partner of IGF2BP1 and contributed to positive regulation of INHBA-Smad2/3 signaling. G3BP1 contains two C-terminal motifs (associated with RNA binding) and an RNA recognition motif (RRM). It has been demonstrated that G3BP1 promotes stress-induced RNA granule interactions to preserve polyadenylated mRNA [45]. Meanwhile, G3BP1 is involved in protein degradation by stably associating with USP10 deubiquitinase [46]. Our experimental results showed that INHBA protein was significantly decreased and IGF2BP1 was slightly downregulated after G3BP1 knockdown. Future studies will be needed to clarify the specific details regarding whether and how the interaction between IGF2BP1 and G3BP1 activate INHBA-Smad2/3 signaling.

Several studies have demonstrated that BTYNB, a structure-specific inhibitor, could block the binding of IGF2BP1 to its oncogenic target mRNA, thus disrupting their interaction [32, 33]. We evaluated the efficacy of BTYNB in vitro and found that the typical malignant phenotypes of ESCC cell lines with high IGF2BP1 expression were sharply repressed, and the IGF2BP1-INHBA interaction was disturbed by BTYNB. Our results implied that IGF2BP1 could be a potential target of ESCC.

As mentioned above, IGF2BP1 has long been considered an oncofetal protein. In fact, according to the HPA database, IGF2BP1 mRNA is expressed only in the testis and placenta and weakly in the kidney, and IGF2BP1 protein is expressed only in the adult testis, ovary, and bronchial tissues, supporting an expression pattern in few adult tissues. In the present study, our IHC results showed that IGF2BP1 is highly expressed in nearly 50% of ESCC tissues but not expressed or only weakly expressed in all surgical margin specimens. Based on these published data and our observations, IGF2BP1 could be a very promising target for ESCC, making it possible to specifically target tumor cells without disturbing noncancerous tissues. These data imply that IGF2BP1 is a potential molecular target for ESCC therapy.

Little information is available describing how the expression of IGF2BP1 is modulated at the transcriptional and posttranscriptional levels. It has been proposed that IGF2BP1 transcription is induced by β-catenin [47] and c-Myc [48]. In addition, let-7 could regulate IGF2BP1 posttranscriptionally [49]. Our observation linked genomic hypomethylation in the first intron to high IGF2BP1 expression, suggesting a new perspective on aberrant transcription of this gene.

## Conclusion

In conclusion, our data demonstrate that upregulation of IGF2BP1 is a frequent event in ESCC tissues and might serve as a candidate biomarker for the disease. The present study reveals for the first time that elevated IGF2BP1 plays a pivotal role in the invasion and migration of ESCC cells by activating the IHNBA-Smad2/3 signaling pathway, providing a promising and attractive target for ESCC patients with high expression of IGF2BP1.

### Electronic supplementary material

Below is the link to the electronic supplementary material.

**Additional file 1: Table S1-7.** Supplementary Materials and Methods.

**Additional file 2: Figure S1**. The read distribution of genes identified by RIP-Seq. A. Distribution of reads on all genes. B. Read distribution across all peak-associated gene functional regions. The graph above shows the cumulative distribution of reads across all functional regions of the genes (total reads are logarithmic base 10). The graph below shows the distribution of reads on each gene, with a gradient in color from blue to yellow to red, representing the coverage depth from shallow to deep.

**Additional file 3: Table S8-10**. Table S8. Potential target genes of IGF2BP1 identified using RIP-seq. Table S9. Candidate genes selected from RIP-seq results. Table S10. Relative mRNA expression of candidate genes after IGF2BP1 knockdown in KYSE30 and TE1 cells.

**Additional file 4: Figure S2**. Gene expression alterations of candidate genes in ESCC cell lines with stable IGF2BP1 knockdown. Abundance of mRNA was determined by real-time RT-PCR normalized to GAPDH mRNA level, and the fold changes of gene expression were shown by heat map. The red squares represent up-regulation in gene expression, while blue represents down-regulated gene expression. AVG: average.

**Additional file 5: Figure S3**. Coverage of reads on INHBA gene in RIP-seq. The horizontal axis shows the gene location, the left vertical axis shows the gene coverage, and the right vertical axis shows the sample name.

**Additional file 6: Table S11-13.** Table S11. Potential interactive proteins of IGF2BP1 identified using MS. Table S12. Proteins identified using MS that regulate RNA. Table S13. Candidate interacting proteins of IGF2BP1 selected from Co-IP-MS.

## Data Availability

The datasets (TCGA.ESCA.sampleMap/HiSeqV2) analyzed during the current study are available in the UCSC Xena TCGA hub repository, https://tcga.xenahubs.net.

## Abbreviations

ESCC: Esophageal squamous cell carcinoma
SCC: Squamous cell carcinoma
HNSCC: Head and neck squamous cell carcinoma
LUSC: Lung squamous cell carcinoma
CESC: Cervical squamous cell carcinoma
IHC: Immunohistochemistry
RISH: RNA in situ hybridization
TMA: Tissue microarray
H&E staining: Haematoxylin & eosin staining
IF: Immunofluorescence
siRNA: small interfering RNA
NC: Negative control
RIP: RNA co-Immunoprecipitation
RT-PCR: Reverse transcription-polymerase chain reaction
WB: Western blotting
Co-IP-MS: Co-immunoprecipitation-mass spectrometry
MSP-PCR: Methylation specific PCR
TCGA: The Cancer Genome Atlas
HPA: Human Protein Atlas
GO: Gene Ontology
KEGG: Kyoto Encyclopedia of Genes and Genomes
RBPs: RNA-binding proteins
IGF2BPs: Insulin-like growth factor-2 mRNA-binding proteins
CDS: Coding sequence
m^6^A: N6-methyladenosine
TGF-β: Transforming growth factorβ
G3BP1: GTPase activating protein (SH3 domain) binding protein 1
RNP: Ribonucleoprotein
RRM: RNA recognition motif
ActD: actinomycin D
CHX: Cycloheximide
