# Supplementary material for: Elevated expression of the RNA-binding protein IGF2BP1 enhances the mRNA stability of INHBA to promote the invasion and migration of esophageal squamous cancer cells
Source: Exp Hematol Oncol. 2023 Aug 29;12:75. doi: 10.1186/s40164-023-00429-8 (PMC10466848; doi:10.1186/s40164-023-00429-8)
Supplement: Supplementary file 1 — Additional file 1: Table S1-7. Supplementary Materials and Methods. [file 40164_2023_429_MOESM1_ESM.docx]

**Supplementary Materials and Methods**

The primer, oligonucleotides and biotin-labeled DNA probes were synthesized by Sangon Biotech (Shanghai) Co., Ltd. And siRNAs were synthesized by Shanghai GenePharma Co., Ltd.

1. **Transient overexpression plasmid constructs**

The coding sequence (CDS) of human INHBA gene was amplified by RT-PCR from ESCC cell lines (primers are listed in Table S1). Then the amplified fragment was inserted into pcDNA3.1-His/myc cloning vector to generate the overexpression construct pcDNA3.1-INHBA. The primers were listed in Supplementary Table S1.

1. **Vector plasmid constructs for lentivirus interference and or lentivirus packaging**

The IGF2BP1 shRNA or sh-Nonsilencing oligonucleotides were annealed to form double strands, and then inserted to pLKO.1-TRC vector to generate the pLKO.1-shIGF2BP1/NS plasmid vector (the oligonucleotides sequences are provided in Table S2). Retroviruses were packaged by transfection of 293FT cells with the retroviral vectors psPAX2 and pMD2G along with pLKO.1-shIGF2BP1/NS.

All constructs were validated by sequencing.

1. **Transfection and lentiviral transduction**

Cells were transfected with siRNA (GenePharma, China) for 48 hours or overexpression plasmids for 24 hours using Lipofectamine 3000 Transfection Reagent (L3000015, Thermo) according to the manufacturer’s instructions. Lentiviruses expressing IGF2BP1-shRNA (shIGF2BP1) or no-silencing-shRNA (shNS) as a negative control were used to infect ESCC cells, and stable cell strains were selected by puromycin (1 μg/mL, Gibco) for one week. The shRNA and siRNA sequences are provided in Supplementary Table S2 and Table S3.

1. **Cellular invasion and migration assays**

24-well Transwell plates with 8 μm pores polycarbonate membrane inserts (3422, Corning, USA) were employed to assess the invasion and migration ability of ESCC cells. Matrigel basement membrane matrix (356234, Corning) was thawed on ice at 4 °C overnight, and diluted in serum-free medium at a ratio of 1:33 (v/v). Then 50 μL of the diluted Matrigel matrix was added to the center of each Transwell insert for invasion assays. The plates were incubated at 37 °C for 1 hour to allow the Matrigel matrix to form a gel. For migration assays, the Transwell inserts were not coated with Matrigel matrix.

Cultured cells were trypsinized and seeded into the upper chambers at a density of 1×10^5^/well in 200 μL of serum-free medium, and 700 μL of RPIM-1640 medium with 30% fetal bovine serum was added to the lower chambers as a chemoattractant. After incubation for 36 hours (KYSE30) or 24 hours (TE1) at 37 °C, the Transwell inserts were washed twice with PBS, fixed with methanol and acetone fixation solution (1:1, v/v), and stained with 0.1% crystal violet (Sigma). The cells on the inside of the inserts were gently removed using moistened cotton swabs. The polycarbonate membranes with stained cells were cut off from the Transwell inserts, placed on slide and mounted with coverslip, followed by scanning and imaging with a Nano Zoomer digital pathology biopsy scanner (HAMAMATSU, Japan). The areas covered by stained cells in three random fields were measured by ImageJ.

1. **Coimmunoprecipitation-based mass spectrometry (Co-IP-MS)**

Nondenaturing lysis buffer (P0013, Beyotime) was employed to isolate the total protein lysate. One microgram of IP antibody or rabbit IgG (B900610, Proteintech) was used to immunoprecipitate 1 μg of total protein overnight at 4 °C with rotation. Subsequently, 50 μL of protein A/G magnetic beads (MedChemExpress) were introduced into the mixture and incubated with rotation for 2 h at 4 °C. The beads were rinsed four times with washing buffer, after which they were eluted in 20 μL of 1× loading buffer and denatured at 100 °C for 10 min. Protein samples were then subjected to WB assay or SDS–PAGE followed by Coomassie staining. Gel pieces were cut off and sent to Shanghai Applied Protein Technology Co. Ltd. for mass spectrometry analysis.

Gel pieces were digested overnight in 12.5 ng/μL trypsin. LC–MS/MS analysis was performed on a Q Exactive mass spectrometer (Thermo Scientific) that was coupled to Easy nLC (Thermo Fisher Scientific). MS/MS spectra were searched using the MASCOT engine (Matrix Science, London, UK; version 2.2) embedded in Proteome Discoverer 1.4 (Thermo Electron, San Jose, CA.) against the UniProt Human database (133549 sequences, download on March 3rd, 2013) and the decoy database. The cutoff of the global false discovery rate (FDR) for peptide and protein identification was set to 0.01.

**Table S1. Primers for overexpression vector construction**

| **Plasmid** | **Primer** | **Sequence (5′to 3′)** |
| --- | --- | --- |
| pcDNA3.1-myc His C-INHBA | Forward | GCCGAATTCATGCCCTTGCTTTGGCTGAG |
| pcDNA3.1-myc His C-INHBA | Reverse | ATTCTCGAGCTGAGCACCCACACTCCTCCAC |

**Table S2. The oligonucleotide sequences of shRNA**

| **Targeted Gene** | **oligo** | **Sequence (5′to 3′)** |
| --- | --- | --- |
| Non-silencing | Forward | CCGGGGATCATCATGCTATGCAGTTCTCGAGAACTGCATAGCATGATGATCCTTTTTTG |
|  | Reverse | AATTCAAAAAAGGATCATCATGCTATGCAGTTCTCGAGAACTGCATAGCATGATGATCC |
| IGF2BP1 | Forward | CCGGCTCCAAAGTTCGTATGGTTATCTCGAGATAACCATACGAACTTTGGAGTTTTTTG |
|  | Reverse | AATTCAAAAAACTCCAAAGTTCGTATGGTTATCTCGAGATAACCATACGAACTTTGGAG |

**Table S3. The target sequences of siRNA**

| **Targeted Gene** | **siRNA/shRNA** | **Sequence (5′to 3′)** |
| --- | --- | --- |
| Negative control | NC | TTCTCCGAACGTGTCACGTTT |
| IGF2BP1 | siRNA-1 | GCAGTGGTGAATGTCACCTAT |
|  | siRNA-2 | CCTGGCCCATAATAACTTTGT |
| G3BP1 | siRNA-1 | AGTGCGAGAACAACGAATAAA |
|  | siRNA-2 | TATGGAAAGAACTCTTCTTAT |
| INHBA | siRNA-1 | GGCAGAAATGAATGAACTTAT |
|  | siRNA-2 | AGGCACTTTCCTACCCAATTA |
| METTL3 | siRNA-1 | GCAAGTATGTTCACTATGAAA |
|  | siRNA-2 | GCCAAGGAACAATCCATTGTT |
| METTL14 | siRNA-1 | CCATGTACTTACAAGCCGATA |
|  | siRNA-2 | GCTAATGTTGACATTGACTTA |

**Table S4. Antibodies for immunoreaction**

| **Name** | **Supplier** | **Cat. #** | **Application** |
| --- | --- | --- | --- |
| Recombinant Anti-IGF2BP1/IMP1 antibody | Abcam | ab184305 | IHC/WB 1:1000 |
| IGF2BP1 Antibody (D-9) | Santa | sc-166344 | IF 1:50 |
| Anti-IGF2BP1 (IMP1) pAb | MBL | RN007P | RIP 1 μg |
| Recombinant Anti-Inhibin beta A antibody | Abcam | ab128958 | WB 1:1000 |
| Smad2/3 (D7G7) XP® Rabbit mAb | CST | 8685T | WB 1:1000 |
| Recombinant Anti-G3BP antibody | Abcam | ab181150 | WB 1:1000/ IF 1:100 |
| Recombinant Anti-METTL3 antibody | Abcam | ab195352 | WB 1:1000 |
| METTL14 Polyclonal antibody | Proteintech | 26158-1-AP | WB 1:500 |
| GAPDH Monoclonal antibody | Proteintech | 60004-1-Ig | WB 1:1000 |
| anti-mouse/rabbit IgG, HRP conjugate | Proteintech | SA00001-1/2 | WB 1:5000 |
| Rabbit IgG | Proteintech | B900610 | Co-IP 1 μg |
| HRP-conjugated IgG Fraction Monoclonal Mouse Anti-Rabbit IgG, Light Chain Specific | Proteintech | SA00001-7L | Co-IP 1:5000 |
| CoraLite488-conjugated Goat Anti-Rabbit IgG(H+L) | Proteintech | SA00013-2 | IF 1:200 |
| CoraLite594-conjugated Goat Anti-Mouse IgG(H+L) | Proteintech | SA00013-3 | IF 1:200 |
| Anti-m^6^A antibody | Millipore | ABE572 | m^6^A-PCR 5μg |

**Table S5. Primers for RT-PCR/qRT-PCR analysis**

| **Gene** | **Primer** | **Sequence (5′to 3′)** |
| --- | --- | --- |
| IGF2BP1 | Forward | GGGCCATCGAGAATTGTTGC |
|  | Reverse | CGGGAGCCTGCATAAAGGAG |
| INHBA | Forward | ACGGGTATGTGGAGATAGAGGA |
|  | Reverse | GGACTTTTAGGAAGAGCCAGACT |
| GAPDH | Forward | GGAGCGAGATCCCTCCAAAAT |
|  | Reverse | GGCTGTTGTCATACTTCTCATGG |

**Table S6. DNA probes for RNA pull-down assay**

| **Gene** | **DNA probe** | **Sequence (5′to 3′)** |
| --- | --- | --- |
| INHBA | Sense | Biotin-GGCAGAAATGAATGAACTTAT |
|  | Antisense | Biotin-ATAAGTTCATTCATTTCTGCC |

**Table S7. Primers for MSP-PCR analysis of *IGF2BP1***

| **Amplification**  **region** | **M/U** | **Primer** | **Sequence (5′to 3′)** |
| --- | --- | --- | --- |
| Site # 1 | Methylated | Forward | GTAGAGTTTTAGGGACGTTGTCG |
|  |  | Reverse | CCGACGCCACAAAACCCCTCG |
|  | Unmethylated | Forward | GTAGAGTTTTAGGGATGTTGTTG |
|  |  | Reverse | CCAACACCACAAAACCCCTCA |
| Site # 2 | Methylated | Forward | CGTCGTGTCGTTCGTTTTTTTGCGCGTC |
|  |  | Reverse | CGCGCAAAAATCGCGAAAAAATTCG |
|  | Unmethylated | Forward | TGTTGTGTTGTTTGTTTTTTTGTGTGTT |
|  |  | Reverse | CACACAAAAATCACAAAAAAATTCA |
| Site # 3 | Methylated | Forward | CGGATCGTTTGCGATTTGTTCGTTTCGC |
|  |  | Reverse | ACCGCCGCGCCACCCCGCTCACG |
|  | Unmethylated | Forward | TGGATTGTTTGTGATTTGTTTGTTTTGT |
|  |  | Reverse | ACCACCACACCACCCCACTCACA |
