# Supplementary figures and images for: Elevated expression of the RNA-binding protein IGF2BP1 enhances the mRNA stability of INHBA to promote the invasion and migration of esophageal squamous cancer cells
Source: Exp Hematol Oncol. 2023 Aug 29;12:75. doi: 10.1186/s40164-023-00429-8 (PMC10466848; doi:10.1186/s40164-023-00429-8)

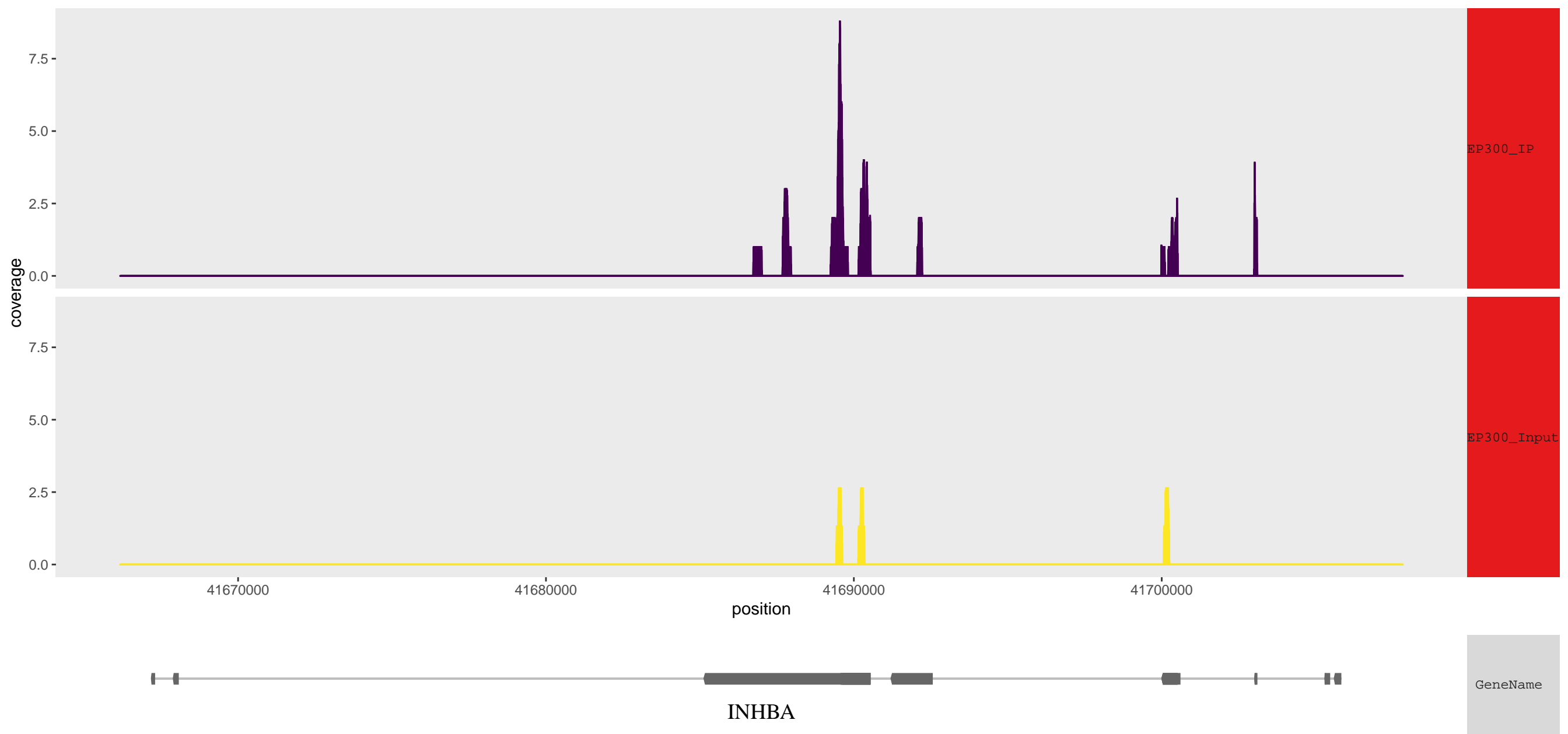

Supplement: Supplementary file 2 — Additional file 2: Figure S1. The read distribution of genes identified by RIP-Seq. A. Distribution of reads on all genes. B. Read distribution across all peak-associated gene functional regions. The graph above shows the cumulative distribution of reads across all functional regions of the genes (total reads are logarithmic base 10). The graph below shows the distribution of reads on each gene, with a gradient in color from blue to yellow to red, representing the coverage depth from shallow to deep. [file 40164_2023_429_MOESM2_ESM.pdf]
